# Lithium europium(III) molybdate(VI), Li_3.5_Eu_1.5_(MoO_4_)_4_

**DOI:** 10.1107/S1600536812000268

**Published:** 2012-01-11

**Authors:** Dan Zhao, Jing Zhu, Peng Liang, Huan Chang, Rong He

**Affiliations:** a Department of Physics and Chemistry, Henan Polytechnic University, Jiaozuo, Henan 454000, People’s Republic of China; b Department of Material Science and Engineering, Yunnan University, Kunming 650091, People’s Republic of China

## Abstract

The title compound, Li_3.5_Eu_1.5_(MoO_4_)_4_, was prepared by solid-state reactions. The fundamental building units of the structure are LiO_4_ polyhedra (site symmetry 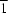), distorted LiO_6_ polyhedra and MoO_4_ tetra­hedra, which are further inter­connected *via* corner-sharing O atoms. One site is occupied by both Li and Eu atoms in a substituent disordered manner (0.25:0.75), and the Li/Eu atoms are coordinated by eight O atoms in a distorted square-antiprismatic manner.

## Related literature

For related rare-earth molybdate compounds, see: Zhao *et al.* (2010 ▶); Ipatova *et al.* (1982 ▶). For similar Li/Eu disorder in LiEu(WO_4_)_2_, see: Chiu *et al.* (2007 ▶).

## Experimental

### 

#### Crystal data

Li_3.5_Eu_1.5_(MoO_4_)_4_
*M* *_r_* = 891.99
Triclinic, 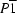

*a* = 5.2182 (11) Å
*b* = 6.7008 (12) Å
*c* = 10.3167 (5) Å
α = 100.09 (2)°
β = 100.341 (15)°
γ = 111.891 (15)°
*V* = 317.49 (9) Å^3^
*Z* = 1
Mo *K*α radiation
μ = 11.22 mm^−1^
*T* = 296 K
0.20 × 0.05 × 0.05 mm

#### Data collection

Rigaku Mercury70 CCD diffractometer
Absorption correction: multi-scan (*ABSCOR*; Higashi, 1995 ▶) *T*_min_ = 0.213, *T*_max_ = 0.604
2478 measured reflections
1437 independent reflections
1224 reflections with *I* > 2σ(*I*)
*R*_int_ = 0.014

#### Refinement

*R*[*F* ^2^ > 2σ(*F* ^2^)] = 0.029
*wR*(*F* ^2^) = 0.079
*S* = 1.10
1437 reflections
105 parameters
Δρ_max_ = 0.95 e Å^−3^
Δρ_min_ = −1.62 e Å^−3^

### 

Data collection: *CrystalClear* (Rigaku, 2004 ▶); cell refinement: *CrystalClear*; data reduction: *CrystalClear*; program(s) used to solve structure: *SHELXS97* (Sheldrick, 2008 ▶); program(s) used to refine structure: *SHELXL97* (Sheldrick, 2008 ▶); molecular graphics: *DIAMOND* (Brandenburg, 2004 ▶); software used to prepare material for publication: *SHELXTL* (Sheldrick, 2008 ▶) and *PLATON* (Spek, 2009) ▶.

## Supplementary Material

Crystal structure: contains datablock(s) I, global. DOI: 10.1107/S1600536812000268/ru2018sup1.cif

Structure factors: contains datablock(s) I. DOI: 10.1107/S1600536812000268/ru2018Isup2.hkl

Additional supplementary materials:  crystallographic information; 3D view; checkCIF report

## Figures and Tables

**Fig. 1.**
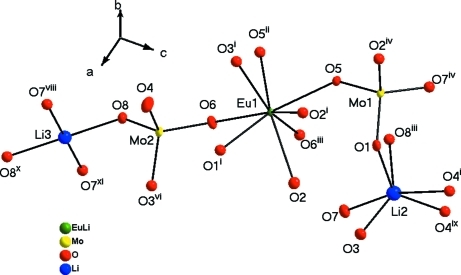
The expanded asymmetric unit of Li3.5Eu1.5(MoO4)2 showing the coordination environments of the Li, Mo and Li/Eu atoms. The displacement ellipsoids are drawn at the 50% probability level.[Symmetry codes: (i) -x + 1, -y + 1, -z + 1; (ii) -x + 1, -y + 2, -z + 1; (iii) -x + 2, -y + 2, -z + 1; (iv) x - 1, y, z; (vi) -x + 2, -y + 1, -z + 1; (viii) x, y, z - 1; (ix) x, y, z + 1; (x) -x + 3, -y + 2, -z; (xi) -x + 3, -y + 2, -z + 1].

**Fig. 2.**
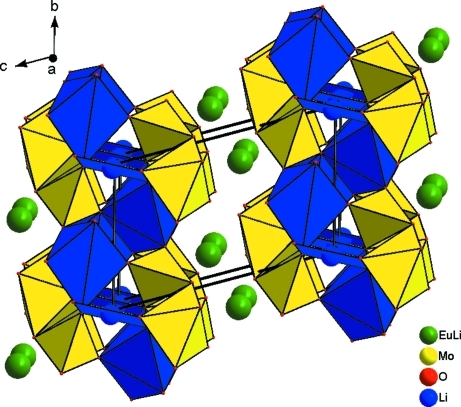
View of the crystal structure of Li3.5Eu1.5(MoO4)2 along [010]. LiO4, LiO6 and MoO4 units are given in the polyhedral representation.

## supplementary crystallographic information

### Comment

In recent years, alkali rare-earth molybdates have been studied mainly due to their rich structural chemistry and interesting physical and chemical properties (Zhao *et al.*, 2010). Of them, a mixed valence alkali rare-earth double molybdate Li_7_Ho_3_(MoO_4_)_8_ (Ipatova *et al.*, 1982) with the substituent disordered structure was reported. In order to enrich this family type of compounds, we report the single-crystal growth and structure investigation of title compound Li_3.5_Eu_1.5_(MoO_4_)_4_.

In this structure, one site is occupancied by both Li and Eu atoms in a substituent disordered manner, denoted as *M* atom, such case can alse be found in compound LiEu(WO_4_)_2_ (Chiu *et al.*, 2007). There are two Li atom sites, two Mo atom sites and one *M* atom site in the asymmetric unit of title compound. Only one Li(3) atom lies on the inversion center in 1 d position, and the other atoms lie on the general positions. One the other hand, the coordination of the two crystallographic distinct Li atoms are different. Li(2) atoms are surround by six O atoms with the bond distances ranging from 1.970 (16) to 2.62 (2) Å, forming distorted LiO_6_ octahedra. Li(3) atoms are surround by four O atoms with the bond distances ranging from 1.968 (4) to 2.046 (4) Å, forming nearly planar LiO_4_ groups. On an over view (Fig. 2), the three-dimensional structure contains LiO_4_ groups, LiO_6_ groups and MoO_4_ tetrahedra, which are further interconnected *via* corner sharing O atoms. The *M* atoms are located on this framework and exhibit a coordination number of eight.

### Experimental

The finely ground reagents Li_2_CO_3_, Eu_2_O_3_ and MoO_3_ were mixed in the molar ratio Li: Eu: Mo = 5: 1: 5, and then placed in a Pt crucible to heat at 573 K for 4 h. The mixture was then re-ground and heated at 1073 K for 20 h, then cooled to 673 K at a rate of 3 K h^-1^, and finally quenched to room temperature. A few colorless crystals of the title compound with prismatic shape were obtained.

### Refinement

The structure contains substitutional disorder in which Li1 and Eu1 occupy the same position. The atomic positional and anisotropic displacement parameters of Li1 and Eu1 atoms were constrained to be identical by using EADP and EXYZ constraint instructions (*SHELXL97*; Sheldrick, 2008). The ratio of Li1 and Eu1 was fixed to 1: 3 to achieve charge balance.

### Crystal data

**Table:**

Li_3.5_Eu_1.5_(MoO_4_)_4_	*Z* = 1
*M**_r_* = 891.99	*F*(000) = 401
Triclinic, *P*1	*D*_x_ = 4.665 Mg m^−^^3^
Hall symbol: -P 1	Mo *K*α radiation, λ = 0.71073 Å
*a* = 5.2182 (11) Å	Cell parameters from 791 reflections
*b* = 6.7008 (12) Å	θ = 2.1–27.5°
*c* = 10.3167 (5) Å	µ = 11.22 mm^−^^1^
α = 100.09 (2)°	*T* = 296 K
β = 100.341 (15)°	Prism, colourless
γ = 111.891 (15)°	0.20 × 0.05 × 0.05 mm
*V* = 317.49 (9) Å^3^	

### Data collection

**Table:**

Rigaku Mercury70 CCD diffractometer	1437 independent reflections
Radiation source: fine-focus sealed tube	1224 reflections with *I* > 2σ(*I*)
graphite	*R*_int_ = 0.014
Detector resolution: 14.6306 pixels mm^-1^	θ_max_ = 27.5°, θ_min_ = 3.4°
ω scans	*h* = −6→6
Absorption correction: multi-scan (*ABSCOR*; Higashi, 1995)	*k* = −8→8
*T*_min_ = 0.213, *T*_max_ = 0.604	*l* = −12→13
2478 measured reflections	

### Refinement

**Table:**

Refinement on *F*^2^	0 restraints
Least-squares matrix: full	Primary atom site location: structure-invariant direct methods
*R*[*F*^2^ > 2σ(*F*^2^)] = 0.029	Secondary atom site location: difference Fourier map
*wR*(*F*^2^) = 0.079	*w* = 1/[σ^2^(*F*_o_^2^) + (0.038*P*)^2^ + 1.5273*P*] where *P* = (*F*_o_^2^ + 2*F*_c_^2^)/3
*S* = 1.10	(Δ/σ)_max_ < 0.001
1437 reflections	Δρ_max_ = 0.95 e Å^−^^3^
105 parameters	Δρ_min_ = −1.61 e Å^−^^3^

### Special details

**Table:**

Geometry. All e.s.d.'s (except the e.s.d. in the dihedral angle between two l.s. planes) are estimated using the full covariance matrix. The cell e.s.d.'s are taken into account individually in the estimation of e.s.d.'s in distances, angles and torsion angles; correlations between e.s.d.'s in cell parameters are only used when they are defined by crystal symmetry. An approximate (isotropic) treatment of cell e.s.d.'s is used for estimating e.s.d.'s involving l.s. planes.
Refinement. Refinement of *F*^2^ against ALL reflections. The weighted *R*-factor *wR* and goodness of fit *S* are based on *F*^2^, conventional *R*-factors *R* are based on *F*, with *F* set to zero for negative *F*^2^. The threshold expression of *F*^2^ > σ(*F*^2^) is used only for calculating *R*-factors(gt) *etc*. and is not relevant to the choice of reflections for refinement. *R*-factors based on *F*^2^ are statistically about twice as large as those based on *F*, and *R*- factors based on ALL data will be even larger.

### Fractional atomic coordinates and isotropic or equivalent isotropic displacement parameters (Å^2^)

**Table:**

	*x*	*y*	*z*	*U*_iso_*/*U*_eq_	Occ. (<1)
Li1	0.62034 (7)	0.77190 (5)	0.43965 (3)	0.00542 (13)	0.25
Eu1	0.62034 (7)	0.77190 (5)	0.43965 (3)	0.00542 (13)	0.75
Mo1	0.16529 (10)	0.70023 (7)	0.68099 (5)	0.00873 (15)	
Mo2	1.08254 (11)	0.84541 (8)	0.19822 (5)	0.01044 (15)	
O1	0.3065 (9)	0.5032 (7)	0.7078 (4)	0.0141 (9)	
O2	0.8045 (9)	0.5637 (7)	0.5724 (4)	0.0145 (9)	
O3	0.7317 (9)	0.3263 (7)	0.7745 (4)	0.0175 (9)	
O4	0.7691 (10)	0.6882 (7)	0.0693 (5)	0.0213 (10)	
O5	0.3812 (8)	0.8834 (6)	0.5948 (4)	0.0099 (8)	
O6	1.0161 (9)	0.9568 (7)	0.3572 (4)	0.0141 (9)	
O7	1.1437 (9)	0.8249 (7)	0.8386 (4)	0.0175 (9)	
O8	1.3010 (9)	1.0575 (7)	0.1374 (4)	0.0167 (9)	
Li2	0.669 (4)	0.593 (3)	0.869 (2)	0.060 (5)*	
Li3	1.5000	1.0000	0.0000	0.049 (6)*	

### Atomic displacement parameters (Å^2^)

**Table:**

	*U*^11^	*U*^22^	*U*^33^	*U*^12^	*U*^13^	*U*^23^
Li1	0.0066 (2)	0.0053 (2)	0.0037 (2)	0.00203 (15)	0.00143 (14)	0.00061 (14)
Eu1	0.0066 (2)	0.0053 (2)	0.0037 (2)	0.00203 (15)	0.00143 (14)	0.00061 (14)
Mo1	0.0097 (3)	0.0099 (3)	0.0068 (3)	0.0038 (2)	0.0029 (2)	0.00267 (19)
Mo2	0.0122 (3)	0.0133 (3)	0.0057 (2)	0.0056 (2)	0.00259 (19)	0.00134 (19)
O1	0.018 (2)	0.016 (2)	0.010 (2)	0.0090 (18)	0.0062 (17)	0.0028 (16)
O2	0.014 (2)	0.012 (2)	0.013 (2)	0.0028 (17)	0.0019 (17)	0.0010 (16)
O3	0.020 (2)	0.019 (2)	0.015 (2)	0.0112 (19)	0.0051 (18)	0.0031 (18)
O4	0.021 (2)	0.021 (2)	0.013 (2)	0.0023 (19)	0.0017 (18)	0.0031 (18)
O5	0.0110 (19)	0.0098 (19)	0.0089 (19)	0.0039 (16)	0.0032 (15)	0.0032 (15)
O6	0.0109 (19)	0.015 (2)	0.014 (2)	0.0028 (17)	0.0057 (17)	0.0009 (17)
O7	0.016 (2)	0.022 (2)	0.014 (2)	0.0081 (19)	0.0044 (17)	0.0013 (18)
O8	0.019 (2)	0.022 (2)	0.014 (2)	0.0114 (19)	0.0082 (18)	0.0083 (18)

### Geometric parameters (Å, °)

**Table:**

Li1—O1^i^	2.368 (4)	O2—Li1^i^	2.464 (4)
Li1—O6	2.370 (4)	O2—Eu1^i^	2.464 (4)
Li1—O5	2.381 (4)	O3—Mo2^vi^	1.786 (4)
Li1—O5^ii^	2.400 (4)	O3—Li2	2.05 (2)
Li1—O3^i^	2.413 (4)	O3—Li1^i^	2.413 (4)
Li1—O2	2.437 (4)	O3—Eu1^i^	2.413 (4)
Li1—O6^iii^	2.442 (4)	O4—Li2^viii^	1.96 (2)
Li1—O2^i^	2.465 (4)	O4—Li2^i^	2.63 (2)
Li1—Li2^i^	3.35 (2)	O5—Eu1^ii^	2.400 (4)
Li1—Eu1^iii^	3.8024 (13)	O5—Li1^ii^	2.400 (4)
Li1—Eu1^ii^	3.8162 (9)	O6—Eu1^iii^	2.442 (4)
Li1—Eu1^i^	3.8915 (10)	O6—Li1^iii^	2.442 (4)
Mo1—O7^iv^	1.737 (4)	O7—Mo1^vii^	1.737 (4)
Mo1—O1	1.772 (4)	O7—Li3^ix^	2.045 (4)
Mo1—O2^iv^	1.799 (4)	O7—Li2	2.50 (2)
Mo1—O5	1.811 (4)	O8—Li3	1.968 (4)
Mo1—Li3^v^	3.3047 (10)	O8—Li2^iii^	2.30 (2)
Mo1—Li2	3.36 (2)	Li2—O4^ix^	1.96 (2)
Mo1—Eu1^iv^	3.6622 (9)	Li2—O8^iii^	2.30 (2)
Mo1—Eu1^ii^	3.7953 (9)	Li2—O4^i^	2.63 (2)
Mo1—Eu1^i^	3.8168 (8)	Li2—Li3^v^	3.314 (19)
Mo2—O4	1.731 (5)	Li2—Li1^i^	3.35 (2)
Mo2—O8	1.753 (4)	Li2—Eu1^i^	3.35 (2)
Mo2—O3^vi^	1.786 (4)	Li3—O8^x^	1.968 (4)
Mo2—O6	1.827 (4)	Li3—O7^xi^	2.045 (4)
Mo2—Li3	3.2648 (7)	Li3—O7^viii^	2.045 (4)
Mo2—Eu1^vii^	3.6433 (9)	Li3—Mo2^x^	3.2648 (7)
Mo2—Eu1^iii^	3.8126 (12)	Li3—Mo1^iii^	3.3046 (10)
O1—Li2	2.10 (2)	Li3—Mo1^xii^	3.3046 (10)
O1—Eu1^i^	2.368 (4)	Li3—Li2^iii^	3.314 (19)
O1—Li1^i^	2.368 (4)	Li3—Li2^xii^	3.314 (19)
O2—Mo1^vii^	1.799 (4)		
			
O1^i^—Li1—O6	72.17 (14)	Mo1—O1—Li1^i^	133.9 (2)
O1^i^—Li1—O5	152.17 (14)	Li2—O1—Li1^i^	97.0 (5)
O6—Li1—O5	135.59 (14)	Eu1^i^—O1—Li1^i^	0.000 (12)
O1^i^—Li1—O5^ii^	128.42 (13)	Mo1^vii^—O2—Li1	118.9 (2)
O6—Li1—O5^ii^	70.23 (13)	Mo1^vii^—O2—Li1^i^	134.1 (2)
O5—Li1—O5^ii^	74.09 (15)	Li1—O2—Li1^i^	105.10 (16)
O1^i^—Li1—O3^i^	75.14 (14)	Mo1^vii^—O2—Eu1^i^	134.1 (2)
O6—Li1—O3^i^	94.56 (15)	Li1—O2—Eu1^i^	105.10 (16)
O5—Li1—O3^i^	100.26 (14)	Li1^i^—O2—Eu1^i^	0.00 (2)
O5^ii^—Li1—O3^i^	74.05 (14)	Mo2^vi^—O3—Li2	143.2 (6)
O1^i^—Li1—O2	70.22 (14)	Mo2^vi^—O3—Li1^i^	119.6 (2)
O6—Li1—O2	101.42 (14)	Li2—O3—Li1^i^	96.9 (6)
O5—Li1—O2	97.18 (13)	Mo2^vi^—O3—Eu1^i^	119.6 (2)
O5^ii^—Li1—O2	151.16 (14)	Li2—O3—Eu1^i^	96.9 (6)
O3^i^—Li1—O2	134.77 (14)	Li1^i^—O3—Eu1^i^	0.00 (3)
O1^i^—Li1—O6^iii^	124.00 (14)	Mo2—O4—Li2^viii^	135.4 (6)
O6—Li1—O6^iii^	75.58 (15)	Mo2—O4—Li2^i^	118.2 (5)
O5—Li1—O6^iii^	71.94 (13)	Li2^viii^—O4—Li2^i^	103.7 (6)
O5^ii^—Li1—O6^iii^	78.18 (14)	Mo1—O5—Li1	123.90 (18)
O3^i^—Li1—O6^iii^	152.23 (14)	Mo1—O5—Eu1^ii^	128.12 (19)
O2—Li1—O6^iii^	72.99 (14)	Li1—O5—Eu1^ii^	105.91 (15)
O1^i^—Li1—O2^i^	79.65 (14)	Mo1—O5—Li1^ii^	128.12 (19)
O6—Li1—O2^i^	150.92 (14)	Li1—O5—Li1^ii^	105.91 (15)
O5—Li1—O2^i^	73.02 (13)	Eu1^ii^—O5—Li1^ii^	0.00 (2)
O5^ii^—Li1—O2^i^	125.81 (14)	Mo2—O6—Li1	125.4 (2)
O3^i^—Li1—O2^i^	70.98 (15)	Mo2—O6—Eu1^iii^	126.0 (2)
O2—Li1—O2^i^	74.91 (16)	Li1—O6—Eu1^iii^	104.42 (15)
O6^iii^—Li1—O2^i^	128.19 (14)	Mo2—O6—Li1^iii^	126.0 (2)
O1^i^—Li1—Li2^i^	38.4 (4)	Li1—O6—Li1^iii^	104.42 (15)
O6—Li1—Li2^i^	87.0 (3)	Eu1^iii^—O6—Li1^iii^	0.000 (17)
O5—Li1—Li2^i^	128.2 (4)	Mo1^vii^—O7—Li3^ix^	121.6 (2)
O5^ii^—Li1—Li2^i^	105.7 (4)	Mo1^vii^—O7—Li2	107.8 (5)
O3^i^—Li1—Li2^i^	37.4 (4)	Li3^ix^—O7—Li2	122.2 (5)
O2—Li1—Li2^i^	101.2 (4)	Mo2—O8—Li3	122.5 (2)
O6^iii^—Li1—Li2^i^	159.8 (3)	Mo2—O8—Li2^iii^	133.5 (5)
O2^i^—Li1—Li2^i^	66.1 (3)	Li3—O8—Li2^iii^	101.5 (5)
O1^i^—Li1—Eu1^iii^	99.68 (11)	O4^ix^—Li2—O3	119.8 (10)
O6—Li1—Eu1^iii^	38.45 (10)	O4^ix^—Li2—O1	136.5 (10)
O5—Li1—Eu1^iii^	104.25 (10)	O3—Li2—O1	89.5 (8)
O5^ii^—Li1—Eu1^iii^	69.98 (10)	O4^ix^—Li2—O8^iii^	89.3 (8)
O3^i^—Li1—Eu1^iii^	128.14 (11)	O3—Li2—O8^iii^	144.5 (10)
O2—Li1—Eu1^iii^	86.31 (10)	O1—Li2—O8^iii^	80.4 (7)
O6^iii^—Li1—Eu1^iii^	37.13 (9)	O4^ix^—Li2—O7	97.9 (8)
O2^i^—Li1—Eu1^iii^	160.33 (10)	O3—Li2—O7	85.3 (7)
Li2^i^—Li1—Eu1^iii^	124.7 (3)	O1—Li2—O7	117.2 (9)
O1^i^—Li1—Eu1^ii^	160.49 (10)	O8^iii^—Li2—O7	69.8 (5)
O6—Li1—Eu1^ii^	103.47 (10)	O4^ix^—Li2—O4^i^	76.3 (6)
O5—Li1—Eu1^ii^	37.21 (9)	O3—Li2—O4^i^	88.5 (7)
O5^ii^—Li1—Eu1^ii^	36.87 (9)	O1—Li2—O4^i^	73.0 (6)
O3^i^—Li1—Eu1^ii^	86.47 (10)	O8^iii^—Li2—O4^i^	120.0 (8)
O2—Li1—Eu1^ii^	128.97 (10)	O7—Li2—O4^i^	168.0 (9)
O6^iii^—Li1—Eu1^ii^	71.20 (10)	O4^ix^—Li2—Li3^v^	64.3 (5)
O2^i^—Li1—Eu1^ii^	100.71 (10)	O3—Li2—Li3^v^	174.0 (9)
Li2^i^—Li1—Eu1^ii^	123.8 (3)	O1—Li2—Li3^v^	84.6 (6)
Eu1^iii^—Li1—Eu1^ii^	86.46 (2)	O8^iii^—Li2—Li3^v^	35.6 (3)
O1^i^—Li1—Eu1^i^	70.99 (10)	O7—Li2—Li3^v^	98.6 (6)
O6—Li1—Eu1^i^	132.65 (10)	O4^i^—Li2—Li3^v^	88.4 (5)
O5—Li1—Eu1^i^	83.87 (9)	O4^ix^—Li2—Li1^i^	156.0 (9)
O5^ii^—Li1—Eu1^i^	156.82 (9)	O3—Li2—Li1^i^	45.7 (4)
O3^i^—Li1—Eu1^i^	103.58 (11)	O1—Li2—Li1^i^	44.6 (4)
O2—Li1—Eu1^i^	37.69 (10)	O8^iii^—Li2—Li1^i^	112.4 (7)
O6^iii^—Li1—Eu1^i^	102.02 (10)	O7—Li2—Li1^i^	99.2 (6)
O2^i^—Li1—Eu1^i^	37.21 (10)	O4^i^—Li2—Li1^i^	83.6 (5)
Li2^i^—Li1—Eu1^i^	82.2 (3)	Li3^v^—Li2—Li1^i^	128.7 (6)
Eu1^iii^—Li1—Eu1^i^	123.78 (2)	O4^ix^—Li2—Eu1^i^	156.0 (9)
Eu1^ii^—Li1—Eu1^i^	120.77 (2)	O3—Li2—Eu1^i^	45.7 (4)
O7^iv^—Mo1—O1	106.9 (2)	O1—Li2—Eu1^i^	44.6 (4)
O7^iv^—Mo1—O2^iv^	106.3 (2)	O8^iii^—Li2—Eu1^i^	112.4 (7)
O1—Mo1—O2^iv^	110.91 (19)	O7—Li2—Eu1^i^	99.2 (6)
O7^iv^—Mo1—O5	116.72 (19)	O4^i^—Li2—Eu1^i^	83.6 (5)
O1—Mo1—O5	108.58 (18)	Li3^v^—Li2—Eu1^i^	128.7 (6)
O2^iv^—Mo1—O5	107.45 (19)	Li1^i^—Li2—Eu1^i^	0.00 (2)
O7^iv^—Mo1—Li3^v^	31.81 (14)	O4^ix^—Li2—Mo1	120.3 (8)
O1—Mo1—Li3^v^	90.06 (14)	O3—Li2—Mo1	115.1 (8)
O2^iv^—Mo1—Li3^v^	138.06 (13)	O1—Li2—Mo1	27.1 (3)
O5—Mo1—Li3^v^	98.83 (13)	O8^iii^—Li2—Mo1	54.5 (4)
O7^iv^—Mo1—Li2	84.1 (4)	O7—Li2—Mo1	108.3 (6)
O1—Mo1—Li2	32.6 (4)	O4^i^—Li2—Mo1	83.6 (5)
O2^iv^—Mo1—Li2	141.4 (4)	Li3^v^—Li2—Mo1	59.4 (3)
O5—Mo1—Li2	100.0 (4)	Li1^i^—Li2—Mo1	69.4 (4)
Li3^v^—Mo1—Li2	59.6 (3)	Eu1^i^—Li2—Mo1	69.4 (4)
O7^iv^—Mo1—Eu1^iv^	102.85 (14)	O8^x^—Li3—O8	180.000 (1)
O1—Mo1—Eu1^iv^	141.46 (14)	O8^x^—Li3—O7^xi^	97.16 (17)
O2^iv^—Mo1—Eu1^iv^	35.65 (13)	O8—Li3—O7^xi^	82.84 (17)
O5—Mo1—Eu1^iv^	78.16 (13)	O8^x^—Li3—O7^viii^	82.84 (17)
Li3^v^—Mo1—Eu1^iv^	127.214 (19)	O8—Li3—O7^viii^	97.16 (17)
Li2—Mo1—Eu1^iv^	173.0 (3)	O7^xi^—Li3—O7^viii^	180.000 (1)
O7^iv^—Mo1—Eu1^ii^	94.98 (15)	O8^x^—Li3—Mo2^x^	26.92 (13)
O1—Mo1—Eu1^ii^	137.51 (14)	O8—Li3—Mo2^x^	153.08 (13)
O2^iv^—Mo1—Eu1^ii^	96.59 (14)	O7^xi^—Li3—Mo2^x^	87.08 (12)
O5—Mo1—Eu1^ii^	29.83 (12)	O7^viii^—Li3—Mo2^x^	92.92 (12)
Li3^v^—Mo1—Eu1^ii^	90.23 (2)	O8^x^—Li3—Mo2	153.08 (13)
Li2—Mo1—Eu1^ii^	119.9 (3)	O8—Li3—Mo2	26.92 (13)
Eu1^iv^—Mo1—Eu1^ii^	61.28 (2)	O7^xi^—Li3—Mo2	92.92 (12)
O7^iv^—Mo1—Eu1^i^	133.27 (15)	O7^viii^—Li3—Mo2	87.08 (12)
O1—Mo1—Eu1^i^	26.58 (13)	Mo2^x^—Li3—Mo2	180.000 (1)
O2^iv^—Mo1—Eu1^i^	95.63 (13)	O8^x^—Li3—Mo1^iii^	123.62 (13)
O5—Mo1—Eu1^i^	94.11 (12)	O8—Li3—Mo1^iii^	56.38 (13)
Li3^v^—Mo1—Eu1^i^	114.76 (2)	O7^xi^—Li3—Mo1^iii^	26.59 (12)
Li2—Mo1—Eu1^i^	55.2 (3)	O7^viii^—Li3—Mo1^iii^	153.41 (12)
Eu1^iv^—Mo1—Eu1^i^	118.02 (2)	Mo2^x^—Li3—Mo1^iii^	109.910 (19)
Eu1^ii^—Mo1—Eu1^i^	123.353 (18)	Mo2—Li3—Mo1^iii^	70.09 (2)
O4—Mo2—O8	107.4 (2)	O8^x^—Li3—Mo1^xii^	56.38 (13)
O4—Mo2—O3^vi^	108.3 (2)	O8—Li3—Mo1^xii^	123.62 (13)
O8—Mo2—O3^vi^	106.3 (2)	O7^xi^—Li3—Mo1^xii^	153.41 (12)
O4—Mo2—O6	112.4 (2)	O7^viii^—Li3—Mo1^xii^	26.59 (12)
O8—Mo2—O6	111.97 (19)	Mo2^x^—Li3—Mo1^xii^	70.090 (19)
O3^vi^—Mo2—O6	110.20 (19)	Mo2—Li3—Mo1^xii^	109.910 (19)
O4—Mo2—Li3	96.73 (15)	Mo1^iii^—Li3—Mo1^xii^	180.0
O8—Mo2—Li3	30.53 (14)	O8^x^—Li3—Li2^iii^	137.1 (4)
O3^vi^—Mo2—Li3	83.23 (14)	O8—Li3—Li2^iii^	42.9 (4)
O6—Mo2—Li3	140.59 (13)	O7^xi^—Li3—Li2^iii^	81.2 (4)
O4—Mo2—Eu1^vii^	140.06 (16)	O7^viii^—Li3—Li2^iii^	98.8 (4)
O8—Mo2—Eu1^vii^	100.92 (14)	Mo2^x^—Li3—Li2^iii^	110.9 (3)
O3^vi^—Mo2—Eu1^vii^	35.15 (14)	Mo2—Li3—Li2^iii^	69.1 (3)
O6—Mo2—Eu1^vii^	80.96 (13)	Mo1^iii^—Li3—Li2^iii^	61.0 (4)
Li3—Mo2—Eu1^vii^	93.60 (2)	Mo1^xii^—Li3—Li2^iii^	119.0 (4)
O4—Mo2—Eu1^iii^	143.13 (16)	O8^x^—Li3—Li2^xii^	42.9 (4)
O8—Mo2—Eu1^iii^	90.70 (14)	O8—Li3—Li2^xii^	137.1 (4)
O3^vi^—Mo2—Eu1^iii^	96.42 (14)	O7^xi^—Li3—Li2^xii^	98.8 (4)
O6—Mo2—Eu1^iii^	31.22 (13)	O7^viii^—Li3—Li2^xii^	81.2 (4)
Li3—Mo2—Eu1^iii^	113.38 (2)	Mo2^x^—Li3—Li2^xii^	69.1 (3)
Eu1^vii^—Mo2—Eu1^iii^	61.522 (18)	Mo2—Li3—Li2^xii^	110.9 (3)
Mo1—O1—Li2	120.3 (6)	Mo1^iii^—Li3—Li2^xii^	119.0 (4)
Mo1—O1—Eu1^i^	133.9 (2)	Mo1^xii^—Li3—Li2^xii^	61.0 (4)
Li2—O1—Eu1^i^	97.0 (5)	Li2^iii^—Li3—Li2^xii^	180.000 (1)

Symmetry codes: (i) −*x*+1, −*y*+1, −*z*+1; (ii) −*x*+1, −*y*+2, −*z*+1; (iii) −*x*+2, −*y*+2, −*z*+1; (iv) *x*−1, *y*, *z*; (v) *x*−1, *y*, *z*+1; (vi) −*x*+2, −*y*+1, −*z*+1; (vii) *x*+1, *y*, *z*; (viii) *x*, *y*, *z*−1; (ix) *x*, *y*, *z*+1; (x) −*x*+3, −*y*+2, −*z*; (xi) −*x*+3, −*y*+2, −*z*+1; (xii) *x*+1, *y*, *z*−1.
